# Impact of surgical timing on wound complications following ankle fracture fixation: A 22-year retrospective cohort study

**DOI:** 10.1016/j.jcot.2026.103405

**Published:** 2026-03-11

**Authors:** Wajiha Zahra, Abdul-Rahman Gomaa, Jubril Babatunde, Mina Seifo, Paul Cool, Simon Pickard

**Affiliations:** a The Robert Jones and Agnes Hunt Orthopaedic Hospital NHS Foundation Trust, Gobowen, Oswestry, UK; b School of Medicine, University of Liverpool, Liverpool, UK; c Royal Wolverhampton NHS Trust, Wolverhampton, UK; d School of Medicine, Keele University, Keele, ST5 5BG, UK; e Royal Shrewsbury Hospital, Shrewsbury and Telford Hospital NHS Trust, Shrewsbury, UK

**Keywords:** Ankle fracture, Surgical timing, Wound complications, Fixation failure, Soft tissue management, Postoperative infection

## Abstract

**Background:**

Ankle fractures are common injuries, with a significant proportion requiring surgical intervention. Despite established treatment principles, the optimal timing for open reduction and internal fixation (ORIF) remains debated. Concerns regarding wound infection risk have led some surgeons to delay fixation, while others advocate for early intervention. This study aimed to assess the impact of surgical timing on postoperative wound infections.

**Methods:**

A retrospective review was conducted on 1130 patients with surgically treated closed ankle fractures at a single institution between 2000 and 2022. Patients were categorised into three groups based on time to surgery: Early (<48 h), Intermediate (48 h to 5 days), and Delayed (>5 days) with timing determined pragmatically according to soft-tissue condition, theatre availability, and both surgeon and patient preference. Postoperative complications, including superficial and deep wound infections, were recorded. The chi-squared test was used to assess associations between surgical timing and infection rates.

**Results:**

There was no significant association between surgical timing and the incidence of superficial wound infections (p = 0.274). However, a significant difference was observed in deep wound infection rates (p = 0.014). Diabetes, particularly Type 1, was significantly associated with both superficial (p < 0.001) and deep infections (p = 0.019). Patients over 60 years had an increased risk of superficial infections (p = 0.016) but not deep infections (p = 0.261). Superficial wound infections required longer hospital stay (p < 0.001) due to the need for intravenous antibiotics and monitoring purposes, while the number of procedures significantly correlated with both superficial (p < 0.001) and deep infections (p < 0.001).

**Conclusion:**

In this study, the timing to surgery did not influence postoperative superficial wound infection rates following ORIF for ankle fractures. However, differences were observed in deep wound infection rates. Patient-related factors including diabetes and age are predictors of wound complications. Length of hospital stay was longer in patients with infections.

**Level of evidence:**

IV

## Graphical abstract

**Figure:**
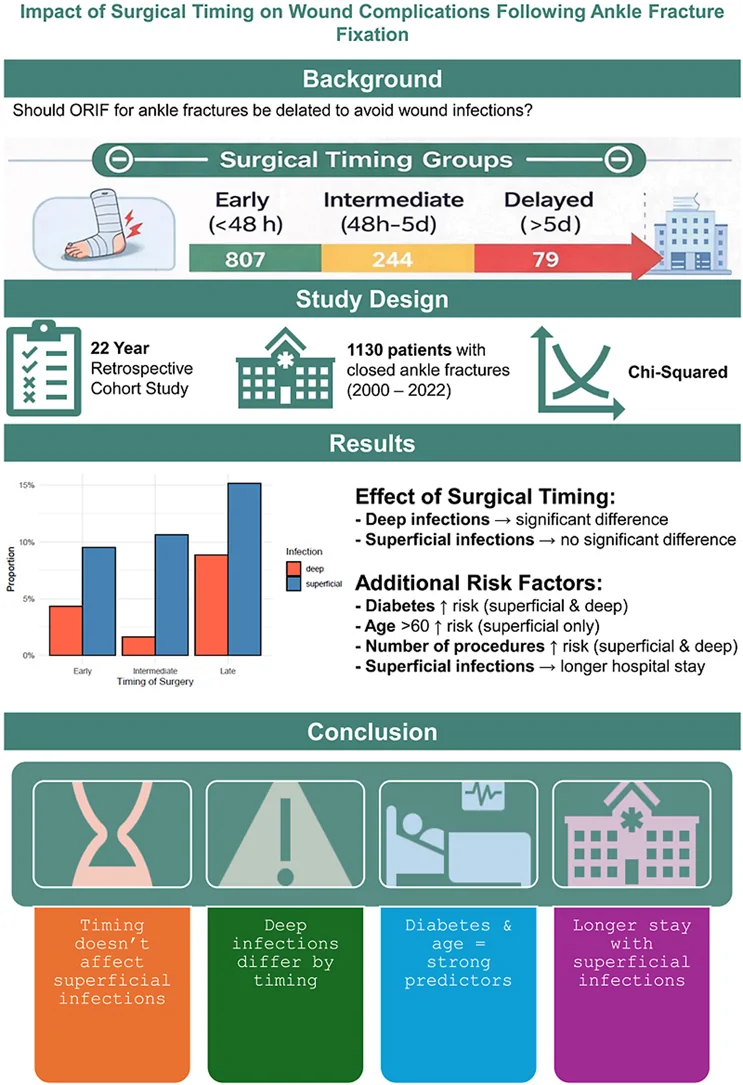

## 1 Introduction

Ankle fractures are common injuries, accounting for approximately 10% of all fractures, with an estimated incidence of 90,000 cases per year in the United Kingdom.^1^, ^2^, ^3^, ^4^, ^5^, ^6^ Approximately 25% to 40% of ankle fractures require surgical management, with the decision often based on the fracture's instability and the patient's overall health.^7^^,^^8^ These fractures affect individuals across all age groups with a tendency towards a bimodal distribution with younger males experiencing a peak up to 20 years of age before declining, while females show an initial peak between 10 and 19 years and a rising incidence beyond 70 years.^9^ High-energy trauma is the predominant cause in younger patients, while low-energy falls are more common among the elderly.^9^^,^^10^ Surgical fixation with open reduction and internal fixation (ORIF) is often required to restore the anatomical congruity of the ankle mortise, ensuring joint stability and optimal functional outcomes. However, one of the most concerning complications following ankle fixation surgery is wound infection, which can lead to metalwork failure, delayed or non-union of the fractures.^7^ Despite well-established principles of fracture management, the optimal timing of surgical intervention remains a topic of controversy. The decision on when to proceed with surgery is a balance between the need for timely reduction and the potential risks associated with early or delayed surgery.

Surgical timing is largely dictated by soft tissue swelling, as excessive tension at incision increases wound complications. Operating too soon risks wound breakdown and infection, prompting many surgeons to delay fixation until swelling subsides, often assessed by the "wrinkle sign".^11^ Severe soft tissue injuries, such as open fractures or contusions, may also require delayed definitive fixation to reduce infection risk, sometimes necessitating temporary external fixation.^12^, ^13^, ^14^ Furthermore, other factors which influence surgical timing include fracture stability, patient health, and logistical factors. Unstable fractures may require early intervention, while conditions like diabetes or immunosuppression necessitate caution.^15^, ^16^, ^17^ The ideal timing for ORIF remains debated, with some studies advocating for early fixation (within 24-48 h) to improve outcomes, while others highlight risks of operating before swelling subsides.^12^^,^^18^ Delayed surgery (beyond 7-12 days) may lower infection risk but can prolong recovery.^13^^,^^14^^,^^19^ A proposed "safe window" within six days balances early intervention with soft tissue recovery, though some research suggests timing has minimal impact if soft tissues are optimal at fixation.^19^, ^20^, ^21^.

Given the ongoing debate regarding the optimal timing of ORIF for ankle fractures, this study aims to investigate the impact of surgical timing on wound complications. By assessing postoperative wound complications in these groups, we seek to provide evidence that may help guide clinical decision-making regarding the safest and most effective timing for ankle fixation surgery.

## 2 Methods

### 2.1 Study design and setting

A retrospective review of all surgically treated ankle fractures within a busy District General Hospital in the United Kingdom between February 2000 and December 2022 was performed.

### 2.2 Patient selection

**Fig. 1:**
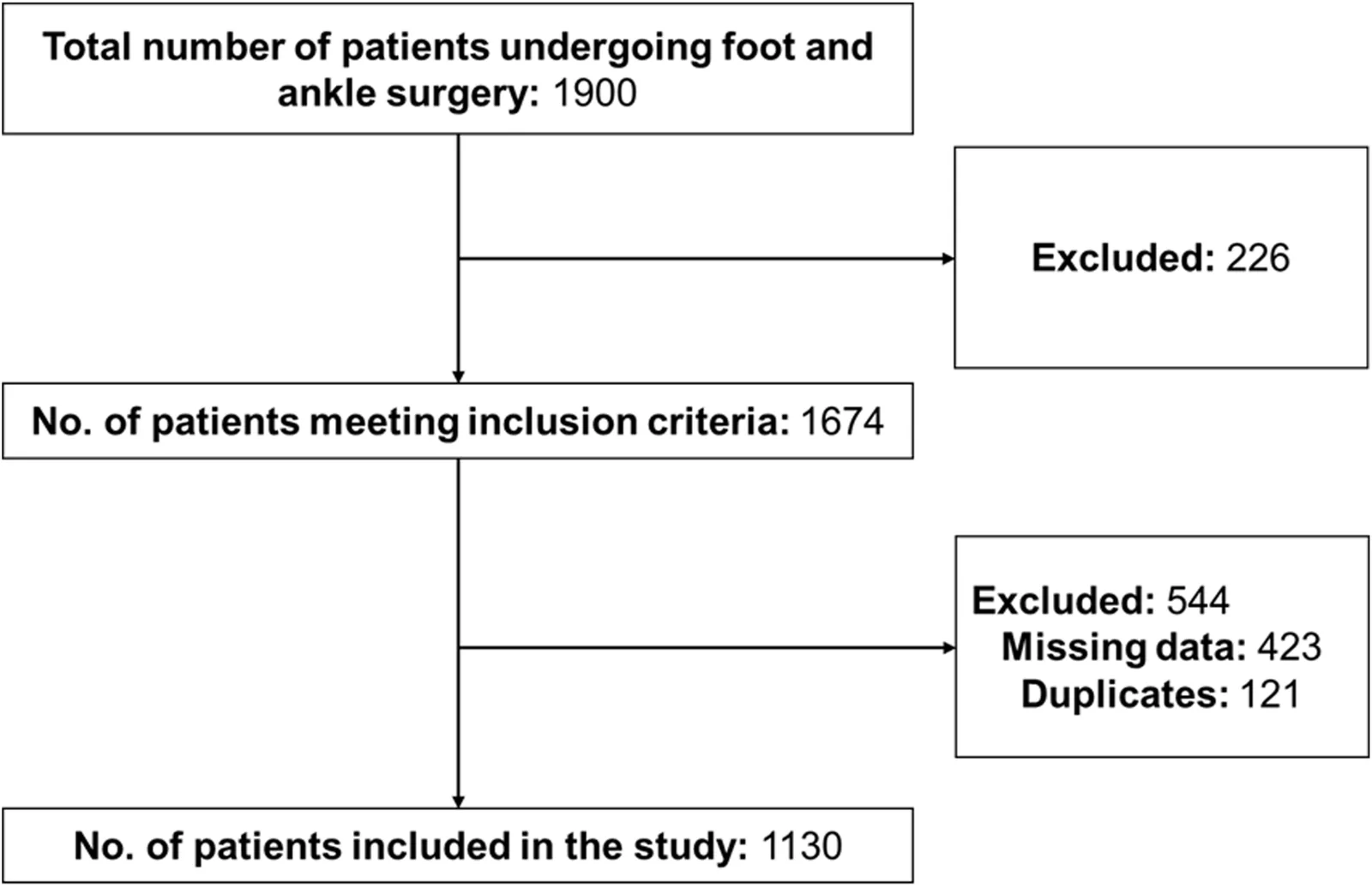
Patient inclusion process from the local ankle fracture database.

Eligible patients included all individuals who underwent surgical treatment for closed ankle fractures and had complete clinical and radiological data. Exclusion criteria were conservatively managed fractures, open ankle fractures, cases requiring initial external fixation (as this is typically associated with factors that independently increase the risk of postoperative complications), and patients with incomplete datasets (Fig. 1).

### 2.3 Data collection

Data collected included patient demographics (age and sex), date of injury, date of surgery, type of injury, details of the surgical procedure, weight-bearing status, postoperative complications, subsequent operative interventions, and the total number of procedures performed, with primary fixation recorded as the first procedure. Mortality data were also captured. All clinical information was obtained from the electronic patient record, and imaging was reviewed via the hospital's Picture Archiving and Communication System (PACS) where available.

### 2.4 Outcome measures

Surgical site infections (SSIs) were classified according to the Centres for Disease Control and Prevention (CDC) criteria.^22^ Superficial SSIs were defined as infections managed non-operatively with antibiotics and wound care, whereas deep SSIs were those requiring surgical intervention, including wound washout, vacuum-assisted closure, or removal of metalwork; confirmed using radiographic and microbiological results. Additional outcome measures included length of hospital stay, the need for further operative procedures, and fixation failure, the latter determined radiologically by evidence of implant failure or delayed/non-union.

### 2.5 Exposure definition

Patients were categorised into three surgical timing groups: Early (<48 h), Intermediate (48 h-5 days), and Delayed (>5 days). Timing was determined pragmatically based on soft-tissue condition, theatre availability, and surgeon and patient preference.

### 2.6 Statistical analysis

Statistical analysis was performed using R Version 4.5.2 (R Core Team, Vienna, Austria). Group characteristics underwent basic descriptive statistical analyses. The Shapiro-Wilk test was used to examine if the data could be modelled with a normal distribution. The chi-squared test was used to compare categorical data. Logistic regression was used to evaluate multivariate associations. P-values less than 5% were considered statistically significant. A priori power analysis was not performed, as the study design was retrospective and involved inclusion of all consecutive eligible cases over the study period rather than predefined sampling.

## 3 Results

### 3.1 Patient characteristics

From an initial database of 1900 ankle fracture cases, 1674 met the inclusion criteria as summarised in Table 1. After removing 121 duplicates and excluding 423 cases with incomplete data, a total of 1130 patients were included in the final cohort. Of these, 500 (44%) were male. The median age was 53 years (IQR: 2.5 years), and the median length of hospital stay was four days (range: 1-131 days; IQR: 5 days). Patients underwent a median of one surgical procedure, with total procedures per patient ranging from one to six. The distribution of patients across timing groups was highly uneven: Early (n = 807), Intermediate (n = 224), and Delayed (n = 79).

**Table 1:** Patient demographics and baseline characteristics.

	Early (within 48 h)	Intermediate (48 h to 5 days)	Delayed (>5 days)
**Total**	807	244	79
**Male: Female**	366 : 441	99 : 145	33 : 46
**Age - years (Mean)**	12 – 98 (51 years)	14 to 90 (54 years)	14 to 87 (57 years)
**>1 Operation**	77 (9.5%)	19 (7.8%%)	14 (17.7%)
**Length of stay (mean)**	24 h to 109 days (4.68 days)	24 h to 81 days (7.87 days)	24 h to 131 days (16 days)

### 3.2 Postoperative wound infections

**Fig. 2:**
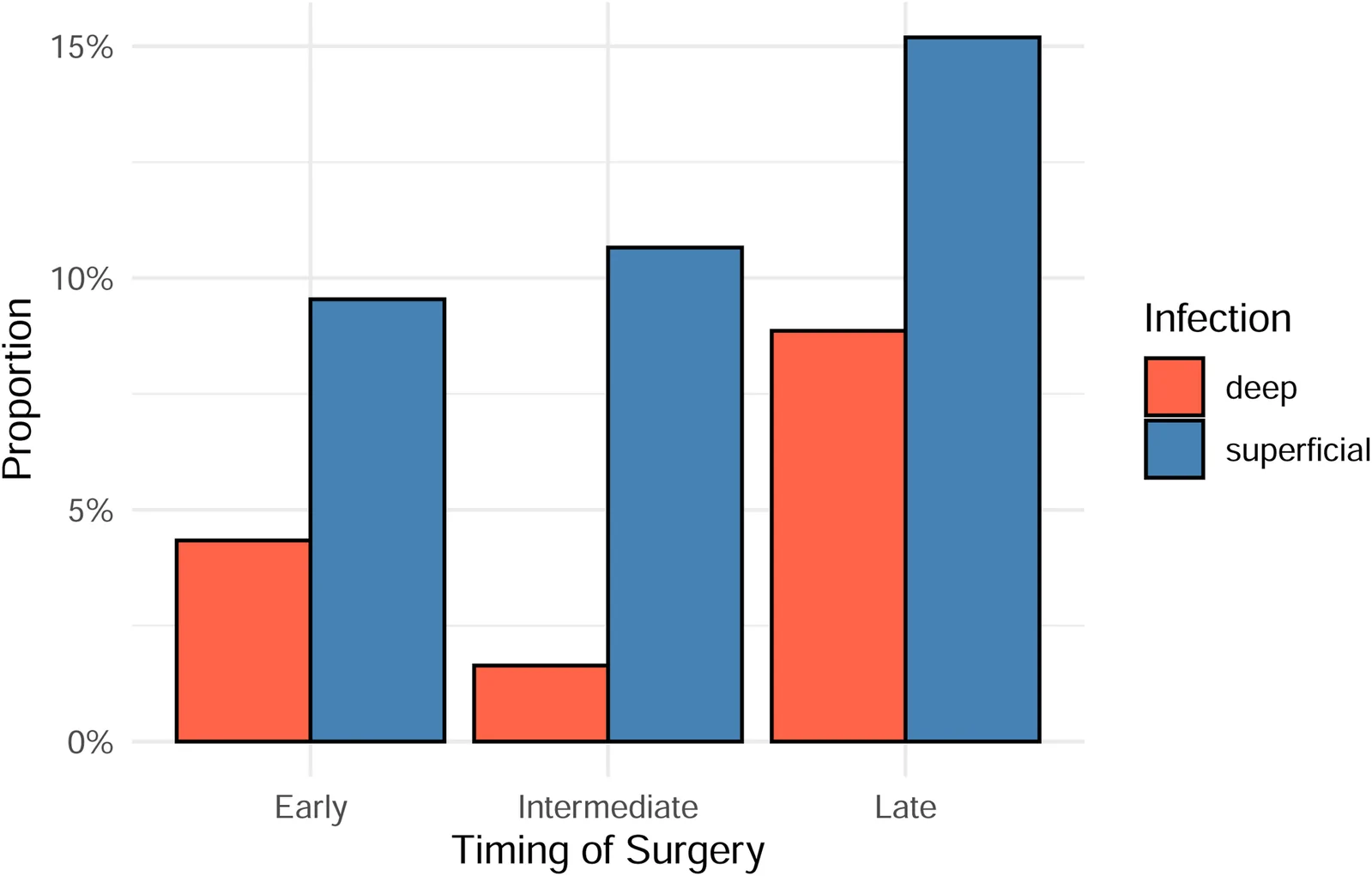
The number of patients developing superficial and deep infections in each group.

The number of patients developing superficial and deep infections in each group are outlined in Table 2 and Fig. 2. There was no significant difference in the incidence of superficial wound infections between the timing groups (early: 9.5%, intermediate: 10.7%, delayed: 15.2%; p = 0.274). However, a statistically significant difference was observed in the rates of deep wound infections across groups (early: 4.3%, intermediate: 1.6%, delayed: 8.9%; p = 0.014).

**Table 2:** The number of patients developing superficial (p = 0.124) and deep (p = 0.014) wound infections based on categorical timing to surgery.

	Superficial Infection (n = 115)		Deep Infection (n = 46)	
	**Yes**	**No**	**Yes**	**No**
**Early** (n = 807)	77 (9.54%)	730 (90.46%)	35 (4.34%)	772 (95.66%)
**Intermediate** (n = 224)	26 (10.66%)	218 (89.34%)	4 (1.64%)	240 (98.36%)
**Late** (n = 79)	12 (15.19%)	67 (84.81%)	7 (8.86%)	72 (91.14%)
**Total** (n = 1130)	115 (10.18%)	1015 (89.82%)	46 (4.07%)	1084 (95.93%)

Superficial infections were associated with a significantly longer hospital stay (p < 0.001). Deep infections strongly correlated with an increased number of surgical procedures (p < 0.001) but were not associated with prolonged admission (p = 0.661).

### 3.3 Risk factors for wound complications

Diabetes was significantly associated with postoperative wound complications. Superficial infections were more common in both Type 1 (p < 0.001) and Type 2 diabetes (p = 0.004), whereas only Type 2 diabetes showed a significant association with deep infections (p = 0.019). Patients over 60 years had a significantly higher likelihood of developing superficial wound infections (p = 0.016), but age was not associated with deep infection (p = 0.261). Sex, fracture laterality, weight-bearing status, peripheral vascular disease, and immunosuppressive therapy were not associated with superficial or deep infections.

### 3.4 Indications for reoperation

Deep wound infection was the leading indication for a second operation across all timing groups. A detailed breakdown of indications for reoperation is provided in Table 3.

**Table 3:** A breakdown of the indications for the second operation.

Indications for second operation	Early (n = 807)	Intermediate (n = 224)	Late (n = 79)
**Metal work removal**	38 (4.71%)	15 (6.70%)	9 (11.39%)
1. Infection	24 (2.97%)	7 (3.13%)	8 (10.13%)
2. Pain	6 (0.74%)	5 (2.23%)	1 (1.27%)
3. Prominent metal work	8 (0.99%)	3 (1.34%)	0 (0%)
**Wound break down**	9 (1.12%)	1 (0.45%)	2 (2.53%)
**Failure of fixation**	7 (0.87%)	1 (0.45%)	1 (1.27%)
**Total**	92 (11.40%)	32 (14.29%)	21 (26.58%)

### 3.5 Logistic regression

Logistic regression confirmed the association with diabetes as a risk factor for superficial (Odds Ratio 3.5, 95% confidence interval 1.4-7.8, p = 0.005) and deep wound infections (Odds Ratio 3.9, 95% confidence interval 1.1-11.1, p = 0.019), but age was not significant (p = 0.083 and p = 0.303) in multivariate analysis. Like univariate analysis, length of stay was associated with superficial and deep infections (Odds Ratio 1.03, 95% confidence interval 1.01-1.05, p = 0.003 and Odds Ratio 1.02, 95% confidence interval 1.00-1.05, p = 0.024). As compared to early surgical intervention, intermediate and late surgical intervention had no association with infection (p = 0.949 and p = 0.961). However, surgery between 48 h and five days (intermediate) was associated with a slightly lower risk of deep infection (Odds Ratio 0.3, 95% confidence interval 0.1-0.8, p = 0.031), but surgery after five days was not significantly different (p = 0.454).

## 4 Discussion

Complications following ankle fracture fixation carry substantial consequences for patient morbidity, long-term functional outcomes, and healthcare costs.^23^, ^24^, ^25^ Among these, surgical site infection is one of the most frequent and clinically significant postoperative complications, with reported incidence ranging from approximately 1.5% to 16% across published cohorts.^26^, ^27^, ^28^ The optimal timing of surgery remains an area of ongoing debate, as concerns persist that early or delayed fixation may influence the risk of infection. Understanding whether early, intermediate, or delayed operative management affects postoperative SSI risk is therefore crucial for informing surgical decision-making and guiding patient counselling. This study aimed to investigate the impact of surgical timing on wound complications. We concluded that timing to surgery did not influence postoperative superficial wound infections; however, a statistically significant difference was observed in deep wound infection rates across timing groups. Conversely, patient-related factors such as diabetes, older age, length of hospital stay, and the number of surgical procedures, were stronger predictors of postoperative wound infections.

In this large retrospective cohort, surgical timing was not associated with the incidence of superficial wound infections, although a statistically significant difference was observed in deep wound infection rates across timing groups. Instead, patient-related factors including diabetes, increasing age, prolonged hospital stay, and a higher number of surgical procedures were more strongly associated with postoperative surgical site infection. These findings suggest that the risk of wound complications depends more on individual patient characteristics and the overall complexity of care rather than the interval between injury and fixation.

Our results are consistent with several contemporary studies and recent reviews. Several adjusted retrospective analyses specifically examining dichotomized early versus delayed windows (under 48 h or within 24 h) report no statistically significant difference after multivariable adjustment.^13^^,^^29^^,^^30^ Tausendfreund et al. highlighted that a broad spectrum of patient, injury, and surgery-related variables such as age, diabetes, smoking, peripheral vascular disease, open fractures and bimalleolar fractures contributed to SSI risk after ankle fracture ORIF.^27^ Delayed surgery was identified as a risk factor in only a minority of studies included within that review.^18^^,^^31^^,^^32^ Furthermore, Hawkins et al. demonstrated that surgical timing did not significantly affect wound complications or union rates, provided fixation was performed within 12 days of injury, and Tantigate et al. likewise found no meaningful difference in wound complications or long-term functional outcomes when comparing early (≤14 days) and delayed (>14 days) surgery.^14^^,^^20^

In contrast, some studies have suggested that delayed surgery may be associated with higher complication rates. Edelstein et al.’s review reported that very early fixation (within 24 h) may reduce wound complications and shorten hospital stay, although the magnitude and consistency of this effect varied across their included studies.^33^ Schepers et al. reported increased infectious wound complications when surgery was delayed beyond one week and identified an 11% complication rate in those undergoing fixation after this threshold.^18^ Their accompanying systematic review also found higher complication rates in delayed cases. Several factors may explain the divergence from our results. First, definitions of “early” and “delayed” fixation vary considerably between studies, making comparisons difficult. Second, earlier work often included more severe fracture-dislocations or used fracture morphology as a proxy for injury severity, variables known to influence complication risk but not consistently captured in all datasets. Third, soft-tissue condition, a principal reason for surgical delay, was frequently not standardised or consistently reported, introducing potential confounding by indication. More recent improvements in implant technology, perioperative optimisation, and infection-prevention strategies may also have attenuated any previously observed relationship between surgical timing and wound complications.

Overall, the current evidence including the findings of this study supports a more individualised, patient-centred approach to determining optimal surgical timing. Rather than adhering to rigid temporal thresholds, clinical decisions should prioritise soft-tissue readiness, comorbidity optimisation, and overall patient condition. Future research must be prospective, multicentred, preferably randomised or at minimum propensity-score matched with granular soft tissue assessment and grading, continuous timing metrics, standardised SSI surveillance, comprehensive comorbidity data to isolate timing effects from confounders with the inclusion of PROMS.

### 4.1 Limitations

This study has several limitations. Its retrospective, single-centre design introduces inherent biases and limits generalisability. A major limitation is the substantial imbalance between timing groups, with most patients undergoing early fixation. This distribution reflects real-world clinical practice where soft-tissue conditions and theatre capacity generally permit early surgery, but it reduces statistical power in the smaller groups and increases susceptibility to confounding by indication.

Important variables known to influence infection risk, including BMI, smoking status, fracture morphology, and mechanism of injury, were not consistently available over the 22-year period and therefore could not be analysed. Differences in these unmeasured factors may have contributed to variation in complication rates independent of surgical timing.

The study is also prone to immortal time bias because surgical timing groups were defined after the decision to operate. Patients in the delayed group must survive until surgery, creating a survivor effect that can distort comparisons of postoperative survival. As survival analysis commenced at the time of surgery rather than injury, these results should be interpreted with caution.

Additionally, the study period encompassed significant changes in implant technology, surgical techniques, and infection-prevention protocols, introducing temporal heterogeneity that could mask associations between timing and outcomes. The absence of propensity matching, multivariable adjustment, and patient-reported outcome measures (PROMs) further limits the ability to draw causal inferences or comment on long-term functional recovery.

## 5 Conclusion

In this large retrospective cohort, surgical timing was not significantly associated with postoperative superficial wound infections following ORIF for ankle fractures; however, a statistically significant association was observed for deep wound infections. Furthermore, patient-related factors particularly diabetes, increasing age, and the need for multiple procedures were also strongly associated with wound complications. Superficial infections were also linked to a prolonged hospital stay.

However, these findings should be interpreted with caution given the study's limitations, including its retrospective design, imbalance between timing groups, incomplete documentation of soft-tissue status, and the absence of several known confounders such as BMI, smoking status, and fracture morphology. The 22-year study period introduces inevitable temporal heterogeneity in surgical practice, implant technology, and perioperative infection-prevention measures. Early in the study period, fixation commonly involved non-locking plates and more extensile approaches, whereas later years saw increasing adoption of locking anatomical implants, minimally invasive techniques, and enhanced perioperative optimisation, including improved glucose control and antibiotic stewardship. These developments may have contributed to lower infection rates in more recent years and could therefore mask or dilute any association between timing to surgery and postoperative wound complications. As our analysis did not stratify outcomes by time, temporal confounding remains an important limitation.

Within these constraints, our results suggest that, once the soft tissues are deemed suitable for surgery, delaying fixation solely to reduce infection risk may not be necessary. Future prospective studies incorporating comprehensive soft-tissue assessment, standardised comorbidity data, and patient-reported outcomes are needed to confirm and refine these findings.

## Patient's consent

Patient consent was not sought as this study was an anonymised retrospective review of patient's care utilising existing medical records.

## Ethics approval

Ethical board review was not needed as this was a retrospective review of case notes. All data were handled in strict accordance with institutional and national information governance and privacy guidelines. Identifiable information was accessed only by authorised personnel, and all analyses were conducted with appropriate safeguards to protect patient confidentiality.

## CRediT author statement

WZ: Data Collection, analysis, paper writing and revision. AG: Paper writing and revision. JB and MS: Data collection. PC: Data collection, analysis, paper writing. SP: Idea, concept and overall supervision.

## Funding

This research did not receive any specific grant funding from agencies in the public, commercial, or not-for-profit sectors.

## Declaration of competing interest

The authors declare the following financial interests/personal relationships which may be considered as potential competing interests:Simon Pickard reports a relationship with KLS Martin Manufacturing LLC that includes: consulting or advisory and speaking and lecture fees. If there are other authors, they declare that they have no known competing financial interests or personal relationships that could have appeared to influence the work reported in this paper.
